# Changes of vaginal microbiota during cervical carcinogenesis in women with human papillomavirus infection

**DOI:** 10.1371/journal.pone.0238705

**Published:** 2020-09-17

**Authors:** Kyeong A. So, Eun Jung Yang, Nae Ry Kim, Sung Ran Hong, Jae-Ho Lee, Chang-Sun Hwang, Seung-Hyuk Shim, Sun Joo Lee, Tae Jin Kim

**Affiliations:** 1 Department of Obstetrics and Gynecology, Konkuk University School of Medicine, Seoul, Republic of Korea; 2 Department of Pathology, CHA Ilsan Medical Center, CHA University, Gyeonggi-do, Republic of Korea; 3 Laboratory of Molecular Oncology, Cheil General Hospital & Women's Healthcare Center, Seoul, Republic of Korea; 4 Human Resource Biobank, Cheil General Hospital & Women's Healthcare Center, Seoul, Republic of Korea; Universidade Estadual de Maringa, BRAZIL

## Abstract

**Objective:**

To evaluate the changes of vaginal microbiota during cervical carcinogenesis in women with high-risk human papillomavirus infection.

**Materials and methods:**

Vaginal microbiota was analyzed using next-generation sequencing in women with normal, cervical intraepithelial neoplasia (CIN), or cervical cancer.

**Results:**

A marked decrease of *Lactobacillus crispatus* was found in the CIN/cancer groups compared with that in the normal group. The diversity of microorganisms increased in patients with CIN or cervical cancer with HPV infection. *Atopobium vaginae* (OR 4.33, 95% CI 1.15–16.32), *Dialister invisus* (OR 4.89, 95% CI 1.20–19.94), *Finegoldia magna* (OR 6.00, 95% CI 1.08–33.27), *Gardnerella vaginalis* (OR 7.43, 95% CI 1.78–31.04), *Prevotella buccalis* (OR 11.00, 95% CI 2.00–60.57), and *Prevotella timonensis* (OR 6.00, 95% CI 1.46–24.69) were significantly associated with the risk of CIN 2/3 or cervical cancer.

**Conclusion:**

Women with the CIN and cervical cancer showed a high diversity in vaginal microbiota. Depletion of *Lactobacillus crispatus* and increased abundance of anaerobic bacteria were detected in women with cervical disease.

## Introduction

Although human papillomavirus (HPV) is an important etiologic agent of cervical carcinogenesis, only a small proportion of women infected by was associated with development of cervical intraepithelial neoplasia (CIN) and cervical cancer [1]. Cervicovaginal environment is thought to be responsible for the development of cervical disease as well as the infection of high-risk HPVs [2]. The vaginal microbiota coexists with various microorganisms in healthy cervicovaginal environment. Predominant *Lactobacillus* species could decrease the pH of the cervicovaginal environment, creating a chemical barrier to the both exogenous bacteria and viruses [3]. The potential role of elevated pH on the vaginal microbiota in cervical carcinogenesis has been reported [4]. In addition, increasing vaginal microbiota diversity has been reported to be associated with progression of CIN disease and may be involved in regulating persistent HPV infection [5, 6].

Recently, next-generation sequencing (NGS) has been applied in clinical research on the vaginal microbiota. NGS method directly amplifies and sequences a particular region of 16S rRNA gene using DNA extracted from the clinical specimen without the need for cell culture [7]. NGS based methods have allowed massive parallelization of bacterial DNA sequencing and facilitated the rapid identification of thousands of vaginal microbiota taxa within a few days [8].

In this study, we examined the composition of vaginal microbiota in women with CIN and cervical cancer, and compared to that normal women using an NGS assay. The effect of high prevalence of specific cervicovaginal bacteria on the risk of developing cervical carcinogenesis in women with high-risk HPV infection was also evaluated.

## Materials and methods

### Specimen collection

A total of 50 cervicovaginal swab specimens were obtained from women aged 20 to 50 at the Department of Obstetrics and Gynecology, Cheil General Hospital and Women’s Healthcare Center. Among the specimens, 40 were positive for high-risk HPV and the remaining 10 were negative for high-risk HPV. All 40 women of high-risk HPV infection were subjected to colposcopic directed cervical biopsy and the histopathologic results were as follows: normal (n = 10), cervical intraepithelial neoplasia (CIN) 1 (n = 10), CIN 2 or 3 (n = 10), and invasive squamous cell carcinoma (n = 10). Ten specimens of negative for high-risk HPV were found to have normal cervical cytology.

### Ethics statement

The study was approved by the Institutional Review Board of Cheil General Hospital (No. CGH-IRB-2017-9). Written informed consent was obtained from all participants. All procedures performed in this study were in accordance with the ethical standards of the institution and with the 1964 Helsinki declaration and its later amendments.

### DNA extraction and NGS

Extraction of DNA from cervicovaginal swab was performed using a DNeasy Blood and Tissue kit (Qiagen, Valencia, CA, USA) according to the manufacturer’s instructions. DNA samples were stored at -20°C. For sequencing purpose, each DNA sample was further prepared according to the Illumina 16S Metagenomic Sequencing Library protocols. Quantification and purity analysis of DNA were conducted using PicoGreen (Thermo Fisher Scientific, Waltham, MA, USA) and Nanodrop (Thermo Fisher Scientific, Waltham, MA, USA), respectively. Genomic DNA (gDNA) was amplified using 16S V3-V4 primers and a subsequent limited‐cycle amplification step was performed for the added multiplexing indices and Illumina sequencing adapters. The 16S rRNA genes were amplified using primers specific to the 16S V3-V4 region. The primer sequences were as follows: Forward primer, 5'-TCGTCGGCAGCGTCAGATGTGTATAAGAGACAGCCTACGGGNGGCWGCAG-3'; reverse primer, 5'-GTCTCGTGGGCTCGGAGATGTGTATAAGAGACAGGACTACHVGGGTATCTAATCC-3'. The final PCR products were normalized and pooled using PicoGreen, and the size of libraries was verified using the TapeStation D1000 DNA ScreenTape (Agilent, USA). The amplified genes were subsequently sequenced using the MiSeq™ platform (Illumina, San Diego, USA).

### Bioinformatic analysis

NGS assay produced a total of 1,809,224 paired end reads, which were analyzed to determine the metagenomic profiles of each sample, calculate intra- and inter-group diversity, and identify differentially represented taxa, using QIIME1. Prior to data analysis, reads were demultiplexed and filtered to remove low-quality data, vector contaminants, and chimeric reads, and two reads in a pair were joined together based decreasing order of overlap scores. Sequence read assignments to operational taxonomic units (OTUs) were performed with QIIME1 using the public database clustered at 99% sequencing identity, and taxonomic frequency profiles were created to reflect the community’s operational taxonomic unit composition at different phylogenetic levels. Rarefaction plots of intra-sample (alpha) diversity were computed for the phylogenetic diversity whole tree metrics. Between groups, beta diversity was calculated using the weighted and unweighted UniFrac similarity measures, and statistical significance was calculated using the analysis of similarity on QIIME1.

### Statistical analyses

Categorical variables were presented as frequencies and percentages. The mean microbial OTUs of normal and cervical disease groups were compared using the nonparametric Mann-Whitney test. The Chi-Square test was used to evaluate the comparison between normal group and CIN 2 or 3/cervical cancer. Associations were shown as odds ratio (OR) with 95% confidence intervals (CI). All p-values of less than 0.05 were considered statistically significant. Data were analyzed using SPSS for Windows (version 17.0; SPSS Inc., Chicago, IL, USA).

## Results

A total of 1,809,244 high-quality reads were analyzed and an average of 69.4 OTUs was observed. Following the removal of singleton OTUs, a total of 478 bacterial species in the vaginal microbiota were identified. Mean numbers of bacterial species were 39.5 in HPV negative normal, 54.9 in HPV positive normal, 68.9 in CIN 1, 59.1 in CIN 2 or 3, 55.7 in cervical cancer groups. There was no difference in the number of bacterial species for HPV negative versus HPV positive in the normal group (p = 0.218). However, more bacterial species were identified in CIN and cervical cancer than that in HPV negative normal (p = 0.039).

Bacterial species with the highest OTUs value in each specimen is shown in Table 1. *Lactobacillus spp*. were the most abundant bacteria with the highest OTUs in normal and CIN 1. However, various other bacteria also showed relatively high OTUs in the specimens from CIN 2 or 3 and cancer.

**Table 1:** The bacteria with the highest operational taxonomic units (OTUs) in each specimen.

HPV- Normal	HPV+ Normal	CIN 1	CIN 2 or 3	Cervical cancer
*Lactobacillus iners* (4)	*Lactobacillus crispatus* (4)	*Lactobacillus iners* (5)	*Lactobacillus iners* (3)	*Fusobacterium necrophorum* (2)
*Lactobacillus crispatus* (3)	*Lactobacillus iners* (1)	*Gardnerella vaginalis* (1)	*Gardnerella vaginalis* (1)	*Gardnerella vaginalis* (1)
*Gardnerella vaginalis* (1)	*Prevotella bivia* (1)	*Sneathia sanguinegens* (1)	*Sneathia sanguinegens* (1)	*Lactobacillus iners* (1)
*Lachnobacterium bovis* (1)	*Prevotella amnii* (1)	*Prevotella timonensis* (1)	*Escherichia fergusonii* (1)	*Escherichia fergusonii* (1)
*Nocardia coeliaca* (1)	*Prevotella timonensis* (1)	*Prevotella buccalis* (1)	*Porphyromonas somerae* (1)	*Lactobacillus crispatus* (1)
	*Streptococcus dysgalactiae* (1)	*Corynebacterium vitaeruminis* (1)	*Atopobium vaginae* (1)	*Streptococcus dysgalactiae* (1)
	*Elizabethkingia miricola* (1)		*Prevotella disiens* (1)	*Haemophilus pittmaniae* (1)
			*Porphyromonas uenonis* (1)	*Parvimonas micra* (1)
				*Peptoniphilus grossensis* (1)

(), number of the samples; HPV, human papillomavirus; CIN, cervical intraepithelial neoplasia.

**Fig 1:**
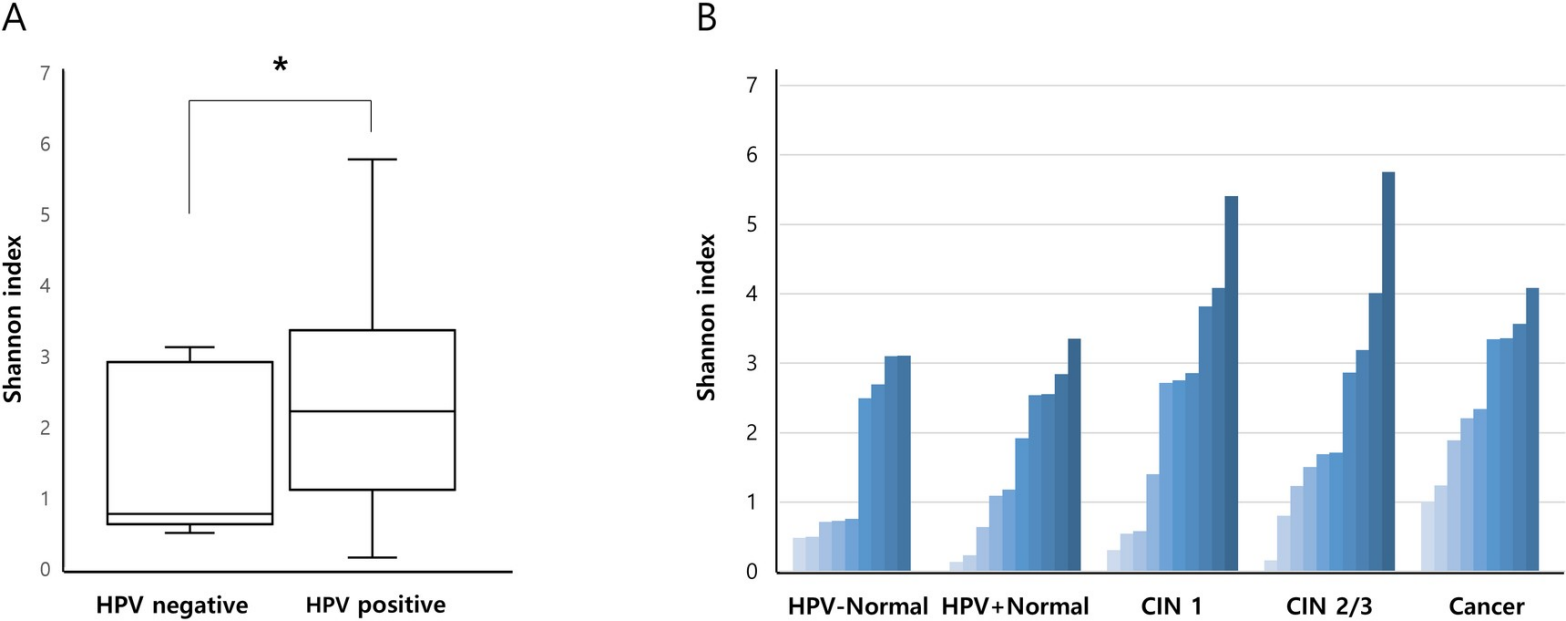
The Shannon diversity index according to the groups. **A.** The mean Shannon diversity index was compared between HPV negative and HPV positive groups. **B.** Shannon diversity index was calculated according to the results of histopathological diagnosis.

Alpha-diversity was calculated for each group (Fig 1). The mean Shannon diversity index was 1.0615 and 2.2297 for HPV negative and HPV positive groups, respectively. The difference between HPV negative and positive groups was statistically significant (p = 0.002) (Fig 1A). The Shannon diversity index showed an increasing trend but it was not significantly associated with the results of histopathological diagnosis (Fig 1B).

**Fig 2:**
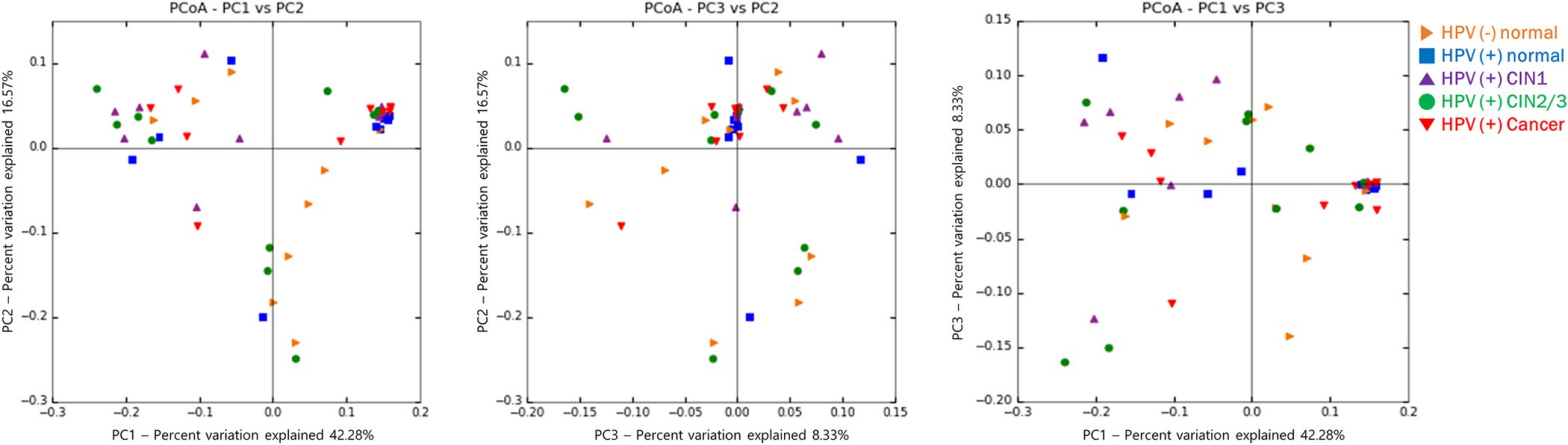
Beta-diversity of microbial communities by histopathological group. The principal coordinate analysis (PCoA) profiles of the groups were displayed in 2D diagram.

The principal coordinate analysis (PCoA) results showed some differences in the vaginal microbiomes of the groups (Fig 2). The three principal coordinates (PCs) were plotted in a 2D diagram showing that PC1 accounted for 42.28%; PC2 accounted for 16.57%; PC3 accounted for 8.33% among the specimens. In the PC1 vs PC2 plot, more than 50% of the HPV-negative normal specimens clustered together in the fourth quadrant, while the majority of the HPV-positive CIN and cancer specimens were in the first and second quadrants but not in the fourth quadrant.

**Fig 3:**
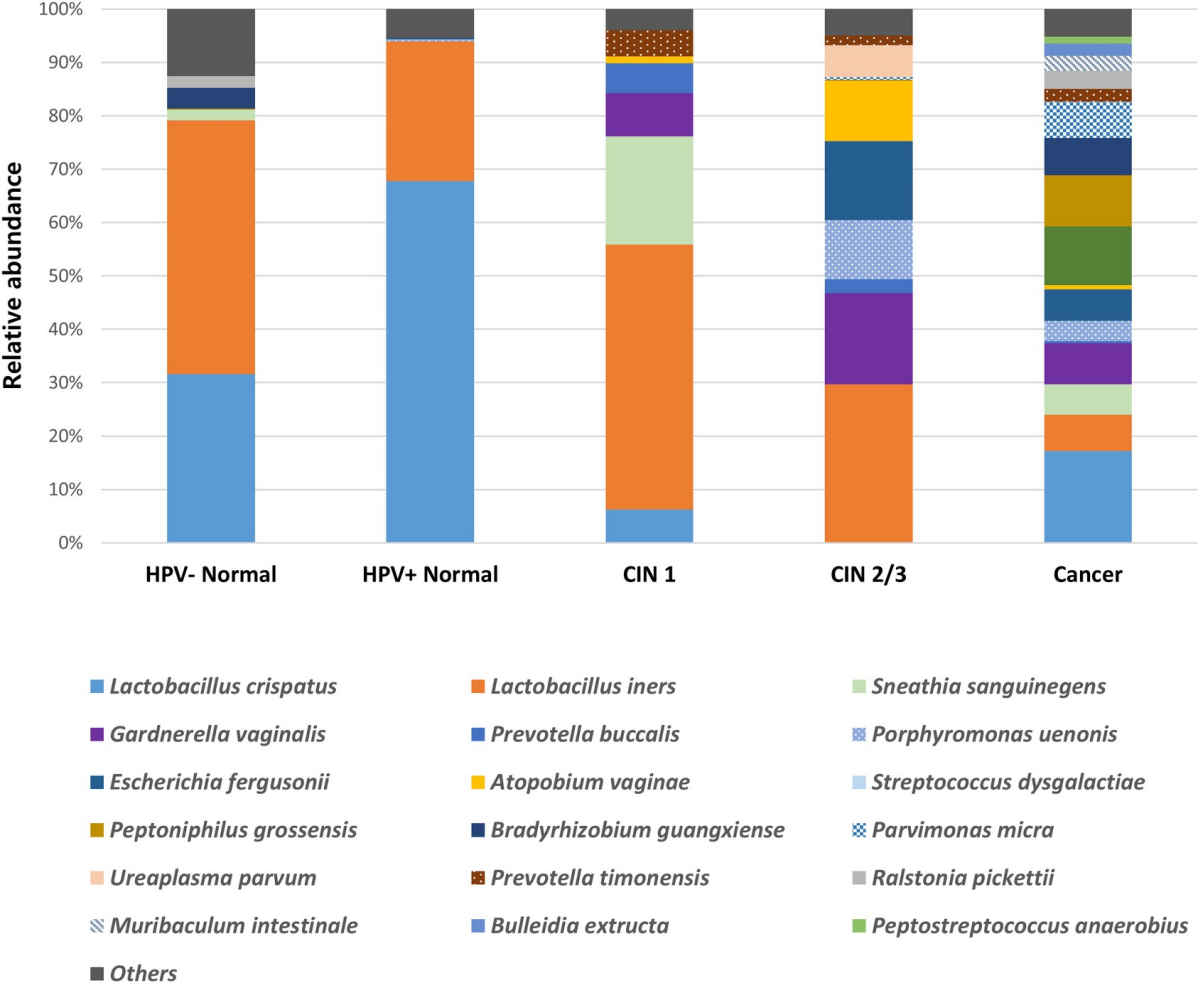
The proportion of operational taxonomic units for the vaginal microbiota, which accounts for more than half in each group. HPV, human papillomavirus; CIN, cervical intraepithelial neoplasia.

To identify clinically significant microbes, sub-analysis was performed on bacterial species detected in more than 50% of each group (HPV negative normal, HPV positive normal, CIN 1, CIN2 or 3, and cancer). A total 56 bacterial species were identified, which accounted for more than half in each group. The relative proportion of the common microbiota in each group is shown in Fig 3. The proportion of *Lactobacillus* spp. decreased in specimens from cervical disease. A marked decrease of *L*. *crispatus* abundance was found in CIN 1, CIN 2 or 3, and cancer compared with that in normal (p = 0.022). However, vaginal microbial diversity was higher in cervical disease group than in the normal group; identified species included *Gardnerella vaginalis* (p = 0.025), *Peptostreptococcus anaerobius* (p = 0.025), and *Porphyromonas uenonis* (p = 0.037).

The species-specific risks for CIN 2 or 3 and cervical cancer are shown in Table 2. Infection by *Atopobium vaginae* (OR 4.33, 95% CI 1.15–16.32), *Dialister invisus* (OR 4.89, 95% CI 1.20–19.94), *Finegoldia magna* (OR 6.00, 95% CI 1.08–33.27), *Gardnerella vaginalis* (OR 7.43, 95% CI 1.78–31.04), *Prevotella buccalis* (OR 11.00, 95% CI 2.00–60.57), and *P*. *timonensis* (OR 6.00, 95% CI 1.46–24.69) were significantly associated with the risk of developing CIN 2 or 3 and cervical cancer.

**Table 2:** The species-specific risk for cervical disease with CIN 2 or 3 and cervical cancer.

	Normal, n (%)	CIN 2or 3/ Cancer, n (%)	Odds ratio (95% CI)	*p*-value
***Atopobium vaginae***				
** Negative**	14 (70)	7 (35)	1	0.027
** Positive**	6 (30)	13(65)	4.33 (1.15–16.32)	
***Dialister invisus***				
** Negative**	16 (80)	9 (45)	1	0.022
** Positive**	4 (20)	11 (55)	4.89 (1.20–19.94)	
***Finegoldia magna***				
** Negative**	18 (90)	12 (60)	1	0.028
** Positive**	2 (10)	8 (40)	6.00 (1.08–33.27)	
***Gardnerella vaginalis***				
** Negative**	13 (65)	4 (20)	1	0.004
** Positive**	7 (35)	16 (80)	7.43 (1.78–31.04)	
***Prevotella buccalis***				
** Negative**	18 (90)	9 (45)	1	0.002
** Positive**	2 (10)	11 (55)	11.00 (2.00–60.57)	
***Prevotella timonensis***				
** Negative**	16 (80)	8 (40)	1	0.010
** Positive**	4 (20)	12 (60)	6.00 (1.46–24.69)	

CIN, cervical intraepithelial neoplasia; CI, confidence intervals.

## Discussion

The development of cervical carcinogensis is dependent on a major HPV-related risk such as virus strain and persistence, viral load, and oncogene expression. Additional risk factors such as smoking, use of oral contraceptives, multiple partners, early onset of sexual activity, and co-infection with other microorganisms have been strongly correlated with cervical carcinogenesis [9]. Co-infection with microorganisms might add further risk of HPV associated carcinogenesis including modification of HPV replication and transcription. In addition, co-infection aggravates inflammation and damage to the epithelium that acts as barrier to HPV infection [10]. Persistent HPV infection is related to imbalanced vaginal microbiota, especially the reduction of lactic acid–producing bacteria. Under the circumstances of *Lactobacillus*-lacking vaginal flora, there is an increased risk of bacterial vaginosis which may upregulate the production of mucin-degrading enzymes but downregulate the production of H_2_O_2_, affecting the production of cytokines, and the susceptibility of cervical mucosa barrier [11].

Changes in the cervicovaginal microbiota involving cervical inflammation and bacterial vaginosis contribute to the progression of HPV infections in high-grade cervical lesions [12]. Healthy vaginal microbiota in the majority of women is dominated by *Lactobacillus* species [13]. In this study we found that the proportion of *Lactobacillus* species were decreased in women with cervical disease, and the abundance of *L*. *crispatus* was significantly reduced in women with CIN and cervical cancer. These results are consistent with previous studies including meta-analyses and systematic reviews [14, 15]. A low abundance of *Lactobacilli* spp. increases the risk of CIN or cervical cancer with a probability of 77.4% and low *Lactobacilli*-dominated community state type (CST) was reported to have two to three times higher risk for developing CIN or cervical cancer compared to *L*. *crispatus*-dominated CST (OR 2.78, 95% CI 1.50–5.16) [14]. Among the *Lactobacillus* species, *L*. *crispatus* are more effective to prevent bacterial dysbiosis than *L*. *iners* [16]. *L*. iners is recognized as a lactobacillus that does not have a protective role in vaginal health. *L*. *crispatus*, but not *L*. *iners*, was associated with the decreased detection of CIN (OR = 0.50, 95% CI = 0.29–0.88) [15]. A relatively low bacterial diversity and high abundance of *Lactobacillus* species has been observed in women with a stable vaginal environment [17]. Loss of *Lactobacillus* dominance promotes the colonization by anaerobic bacterial species with an increase in microbial diversity and increases the risk for cervicovaginal disease [18]. However, the role of *Lactobacillus* species and its association with high-risk HPV infection in the cervical carcinogenesis remain unclear. The changes in vaginal microbiota may be a co-factor for HPV infection.

The association between changes in the composition of vaginal microbiota and HPV infection have been studied extensively, including meta-analyses [19, 20]. The diversity of vaginal microbiota was higher in women with CIN and cervical cancer compared to normal women in this study. In addition, enrichment of anaerobic bacteria such as *Gardnerella vaginalis*, *Peptostreptococcus anaerobius*, and *Porphyromonas uenonis* were detected in women with CIN and cervical cancer. *Gardnerella vaginalis* is a gram-variable facultative anaerobe, and its abundance in the vaginal environment increases dramatically during bacterial vaginosis [21]. *Gardnerella vaginalis* has been reported as a risk factor for cervical disease (OR = 10.17, 95% CI = 3.64–28.41) [22]. *Peptostreptococcus anaerobius* is a gram-positive anaerobic coccus involved in female genital tract infections such as bacterial vaginosis and pelvic inflammatory disease [23] as well as high-grade cervical lesions (p < 0.05) [5]. *Porphyromonas uenonis*, a gram-negative anaerobes, is mainly found in the gastrointestinal, oral cavity, and genital tracts [24]. However, the significance of its association with cervical disease is still unknown.

Among the 56 common bacterial species, infections of *Atopobium vaginae*, *Dialister invisus*, *Finegoldia magna*, *Gardnerella vaginalis*, *Prevotella buccalis*, and *P*. *timonensis* were significantly associated with the risk for high-grade squamous intraepithelial neoplasia and cervical cancer in this study. *Atopobium vaginae* is highly specific for bacterial vaginosis, similarly to *Gardnerella vaginalis*, and enrichment of *Atopobium vaginae* is associated with CIN3 [19, 25]. Dominance of *Atopobium vaginae* in vaginal microbiota represents an important risk factor for development of cervical neoplasia [26]. *Dialister invisus* is a gram-negative coccobacillus and is related with new HPV-type infection within a year in women with normal cytological results [27]. *Finegoldia magna* usually appears on the skin and mucous membranes and is associated with vaginosis, as well as wound infections, soft tissue abscesses, bone infections, and infectious endocarditis [28]. *Prevotella buccalis* and *P*. *timonensis* are belonged to the genus *Prevotella*, gram-negative anaerobic rods which have been isolated from the upper respiratory tract, oral cavity, and urogenital tract [29]. Abundance of *Prevotella* is associated with HPV persistence and inversely related to the abundance of *Lactobacillus* [30].

Vaginal microbiota plays an important role in the health of female genital tract as a defense barrier against vaginal infection. Several studies have reported on vaginal microbiomes in various ethnic groups and there are significant differences in the vaginal microbiota among races. A North American study showed that the proportions of vaginal bacterial communities in asymptomatic women were significantly different among the four ethnic groups including White, Asian, Black, and Hispanic [13]. Vaginal bacterial communities dominated by *Lactobacillus* were found in 80.2% and 89.7% of Asian and white women, respectively, but in only 59.6% and 61.9% of Hispanic and black women, respectively. Another study on Japanese, Caucasian, and Black women found that communities dominated by *Lactobacillus* were common in Japanese and White women, but rare in Black women [31]. These results suggest that the composition of the vaginal microbiota is affected by genetic and cultural background as well as lifestyle.

This study has several limitations. Firstly, we could not show the burden of possible cofactors like smoking, hormones and STDs on the progression of cervical lesions. Secondly, the study was performed based on the data in a retrospective fashion and using a relatively small sample size. Future studies should include prospective data and a larger sample size.

## Conclusions

Although we were not able to fully elucidate the relationship between the change of microbiota and cervical carcinogenesis, we observed significant changes in the vaginal microbial environment in women with CIN and cervical cancer. The depletion of *L*. *crispatus* and increased abundance in anaerobic bacteria such as *Gardnerella vaginalis*, *Peptostreptococcus anaerobius*, and *Porphyromonas uenonis* was significantly more common in women with CIN and cervical cancer. Among them *G*. *vaginalis* was a high risk for developing CIN 2 or 3 and cervical cancer. Therefore, there is a strong association between changes in vaginal microbiota and persistent HPV infection, and improving vaginal microbial environment would reduce the risk of developing cervical cancer.

## Supporting information

S1 Data (XLSX)
